# Precise Thermoplastic Processing of Graphene Oxide Layered Solid by Polymer Intercalation

**DOI:** 10.1007/s40820-021-00755-8

**Published:** 2021-12-04

**Authors:** Zeshen Li, Fan Guo, Kai Pang, Jiahao Lin, Qiang Gao, Yance Chen, Dan Chang, Ya Wang, Senping Liu, Yi Han, Yingjun Liu, Zhen Xu, Chao Gao

**Affiliations:** 1 MOE Key Laboratory of Macromolecular Synthesis and Functionalization, Department of Polymer Science and Engineering, Key Laboratory of Adsorption and Separation Materials & Technologies of Zhejiang Province, Zhejiang University, 38 Zheda Road, Hangzhou, 310027 People’s Republic of China; 2 National Special Superfine Powder Engineering Research Center, Nanjing University of Science and Technology, 1 Guanghua Road, Nanjing, 210094 People’s Republic of China; 3 School of Mechanical Engineering, Southeast University, Nanjing, 211189 People’s Republic of China; 4 Hangzhou Gaoxi Technology Co. Ltd, Yuhang District, Liangzhu, 311113 People’s Republic of China

**Keywords:** Thermoplastic forming, Graphene materials, Polymer intercalation, Processing capability, Structural design

## Abstract

**Abstract:**

The processing capability is vital for the wide applications of materials to forge structures as-demand. Graphene-based macroscopic materials have shown excellent mechanical and functional properties. However, different from usual polymers and metals, graphene solids exhibit limited deformability and processibility for precise forming. Here, we present a precise thermoplastic forming of graphene materials by polymer intercalation from graphene oxide (GO) precursor. The intercalated polymer enables the thermoplasticity of GO solids by thermally activated motion of polymer chains. We detect a critical minimum containing of intercalated polymer that can expand the interlayer spacing exceeding 1.4 nm to activate thermoplasticity, which becomes the criteria for thermal plastic forming of GO solids. By thermoplastic forming, the flat GO-composite films are forged to Gaussian curved shapes and imprinted to have surface relief patterns with size precision down to 360 nm. The plastic-formed structures maintain the structural integration with outstanding electrical (3.07 × 10^5^ S m^−1^) and thermal conductivity (745.65 W m^−1^ K^−1^) after removal of polymers. The thermoplastic strategy greatly extends the forming capability of GO materials and other layered materials and promises versatile structural designs for more broad applications.

**Graphical abstract:**

**Supplementary Information:**

The online version contains supplementary material available at 10.1007/s40820-021-00755-8.

## Highlights

- A solvent-free thermoplastic forming processing of graphene materials is invented by polymer intercalation from graphene oxide precursor.
- The correlation between interlayer spacing and thermoplastic forming capability of polymer-intercalated graphene oxide solid is uncovered.
- The multi-scale forming of graphene materials from Gaussian curved shapes to surface relief patterns with size precision down to 360 nm is realized.

**Supplementary Information:**

The online version contains supplementary material available at 10.1007/s40820-021-00755-8.

## Graphical abstract

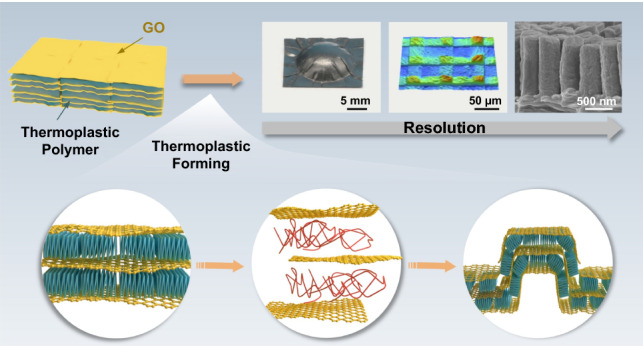

## Introduction

Graphene macroscopic materials have exhibited attractive properties and promised wide applications in structural components, thermal management, sensors and electronic devices [1–4]. To satisfy the demands of more diverse applications of graphene, versatile forming methods are in need in order to design and fabricate precise shapes and structures. The available processing of graphene has relied on the solvent mediated processing methods, including dilute-solution assembly and solvent-plasticized forming. In the former method, dilute dispersions of graphene and its derivates are processed through solution casting [5–7], infiltration [8, 9], spray coating [10, 11] and wet-spinning [12–14], generating diverse forms, such as coatings, films and fibers. The dilute-solution processing has limited structural precision because of the severely volume shrinkage during solution evaporation. On the other hand, a hydroplastic forming method was newly developed to improve the structural precision by decreasing solvent content, introducing a near-solid processing state with solid content of 20–50% [15–17]. As to the extreme cases, solvent-free thermoplastic processing has been utilized to fabricate polymeric and metallic products in the industry [18–21]. For the environmental-friendly merit to avoid solvents and high processing precision for small volume change of thermoplastic process, it is an ultimate pursuit to develop the thermoplastic forming of graphene beyond dilute solution processing.

Thermoplastic processing of graphene is forbidden by its extremely high glass-transition point up to 6300 K, which exceeds the decomposition temperature of graphene (*T*_*d*_) [10]. To achieve the thermoplastic processing of graphene, an intuitive deduction is to blend graphene with the intrinsic thermoplastic polymer component. For this polymer-rich processing range, previous reports on graphene-polymer composites (usually less than 40% of graphene) seem to have minor guidance to the aimed thermoplastic processing of graphene [22–28]. In these polymer-based materials, thermoplastic behavior of polymer matrix is invariably maintained [23, 29]. By contrast, in the graphene-rich range, the polymer exists in a confined state [30, 31]. The strongly confined polymers have been investigated to reveal rich and variable thermodynamic behaviors in many model systems, including absorbed surface, ultrathin self-standing films and trapped pores [32]. However, the thermal motion of confined polymers in the two-dimensional gallery and the consequent thermoplastic behavior of intercalated graphene oxide layered solid remains unclear.

Here, we demonstrate the solvent-free thermoplastic forming of graphene oxide solids by polymer intercalation, achieving the size precision from macro- to sub-micrometer. The thermal transition of intercalated polymer endows the bulk composite film with plasticity. We reveal that the glass-transition temperature (*T*_*g*_) starts to appear and the rigid polymer-intercalated GO solid (Pi-GOS) is able to transform into plasticity when the interlayer spacing exceeds 1.4 nm, indicating a thermal forming criterion of containing above 45%. In the thermoplastic range, we forge flat films into stereoscopic structures with different Gaussian curvatures. We develop a thermoplastic imprinting method to design multiscale surface patterns and periodic arrays in a broad size precision from 360 nm to 200 μm. The thermoplastic imprinting allows the modulation of properties, including Janus material for actuators and responsive film surface with voltage-dependent wetting. Despite of the introduction of polymer plasticizer, the thermoplastic forming materials remain similar electrical (3.07 × 10^5^ S m^−1^) and thermal conductivity (745.65 W m^−1^ K^−1^) as raw films after thermal annealing. The solvent-free thermoplastic forming strategy extends polymer-like forming techniques to graphene and other layered solids and meets complex structural designs in broader applications including humidity sensor, thermal management and functional surfaces.

## Experimental Section

### Materials

Aqueous graphene oxide dispersion (GO, 10 mg mL^−1^) was acquired from Hangzhou Gaoxi Technology Co. Ltd (www.gaoxitech.com). Poly (vinyl alcohol) (PVA, 88% hydrolyzed, average MW 88000) was received from ACROS Organics Co. Ltd. Polyethylene glycol (PEG, 4000 MW) and polyvinylpyrrolidone (PVP, 10000 MW) were purchased from Rhawn.

### Preparation of Pi-GOS

PVA was dispersed into deionized water and the mixture was heated to 60 °C with magnetic stirring for 12 h to prepare solution with a concentration of 1%. Then, PVA solutions were mixed with GO dispersions which is diluted into 5 mg mL^−1^. With a fixed GO mass of 0.1 g, we prepared five solutions with different GO and PVA concentrations: (1) 80 wt.% GO with 20 wt.% PVA; (2) 67 wt.% GO with 33 wt.% PVA; (3) 50 wt.% GO with 50 wt.% PVA; (4) 33 wt.% GO with 67 wt.% PVA and (5) 20 wt.% GO with 80 wt.% PVA. Pi-GOSs were prepared by casting the GO/PVA complex solutions on a polyethylene terephthalate (PET) substrate and the drying process was maintained at the ambient temperature.

### Forming Process

For macro- and micro-scale forming, the Pi-GOS was placed on or between the metallic moulds with designed structures, and then pressed at the forming temperature of 95 °C for varied time. After cooling to room temperature, the formed solid was easily separated from die. For the nanoimprinting process, we chose AAO membrane as template with diameter for 13 mm and hole size for 360 nm. After molding processing, the whole disc was reduced by hydriodic acid at 95 °C for 12 h and immersed in phosphoric acid solution (H_3_PO_4_, 50 wt.%) at 60 °C for 60 min to remove AAO film. To clean off the residual aqueous solution on the surface of disc, the demolded sample was washed by ethanol and n-hexane successively.

### Characterization

The molding processing was carried out on a press (GT-7014-A50C). X-ray diffraction (XRD) profiles were collected on a X`Pert Pro (PANalytical) diffractometer using monochromatic Cu 17 Kα1 radiation (λ = 1.5406 Å) at 40 kV. The glass-transition temperature was investigated by differential scanning calorimetry (DSC) using a TA Q20 instrument. Mechanical tests were taken on TA Q800 at a loading rate of 1 mm min^−1^. The dynamic mechanical analysis was performed on a DMA 242E (NETZSCH Instruments). The measurements were carried out at a frequency of 1 Hz and a temperature ramp of 5 °C min^−1^. 3D-profile images were performed using an optical profilometer (Wyko NT9100). The indentation test was carried out by an Instron 2344 equipped with a load cell. The shells were placed on a plat and stiff base pressed by an indentor at the constant speed of 10 μm s^−1^. Scanning electron microscope (SEM) images were taken on Hitachi S4800 and SU8010 field-emission SEM system. Transmission electron microscope (TEM) images of nanotubes were taken on a HT-7700 HR-TEM. Contact angle measurements were conducted by a video-based, contact angle measuring device (OCA 20).

## Results and Discussion

### Thermoplastic Processing of Pi-GOS

**Fig. 1:**
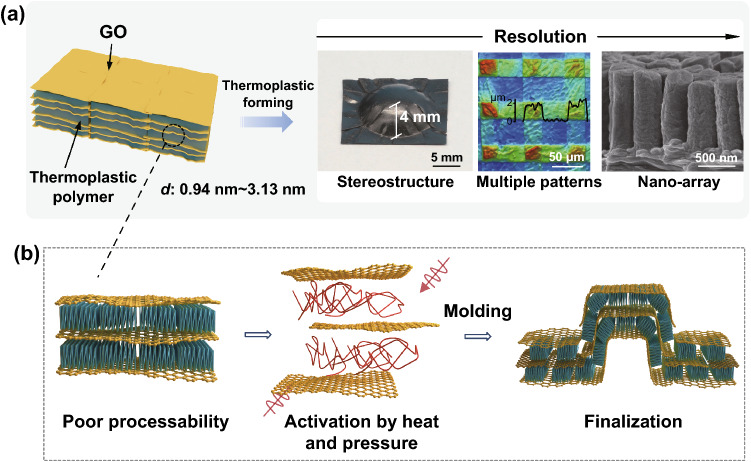
**a** The thermoplastic forming process of Pi-GOS from flat films into different embossed structures ranging from macro- to nano-scale. **b** Schematic diagram of the thermoplastic mechanism, including the sliding of GO sheets and the activation of polymer chains under pressure and heating

The strong interlayer interactions of graphene oxide solid hamper the free motion of graphene oxide layers. Given this, thermoplastic polymer was intercalated between GO layers for the reduction of interlayer van der Waals force, as well as the introduction of thermal response [33–36]. The intercalated polymers should have appropriate glass-transition temperature (*T*_*g*_ < *T*_*d*_) and good affinity to GO, so the variety is constrained. To enable the thermoplastic forming of GO, we introduced polyvinyl alcohol (PVA) into GO solution and prepared homogenously mixed solid papers as raw material for thermoplastic forming (Figs. S1 and S2). The illustration of polymer intercalating process is shown in Fig. S3. The polymeric plasticizers can extend to other species with appropriate *T*_*g*_, like Polyethylene glycol (PEG, *T*_*g*_ ~ 50 °C) and polyvinylpyrrolidone (PVP, *T*_*g*_ ~ 130 °C) (Fig. S4). The film is thermally compressed against template and specific patterns with high precision are formed by the solids creeping into the hole of mold (Fig. 1a, b). When the Pi-GOS is heated to a target temperature (above *T*_*g*_), the polymer segments in the interlamination begin able to move, thus endowing the plasticity of whole material. By thermoplastic processing on Pi-GOS, structures with large scale spanning from 4 to 390 nm were fabricated, including origami with stereo-structures, multiple patterns and nanopillar arrays.

### Thermoplastic Behavior of Intercalated Polymer between GO Sheets

**Fig. 2:**
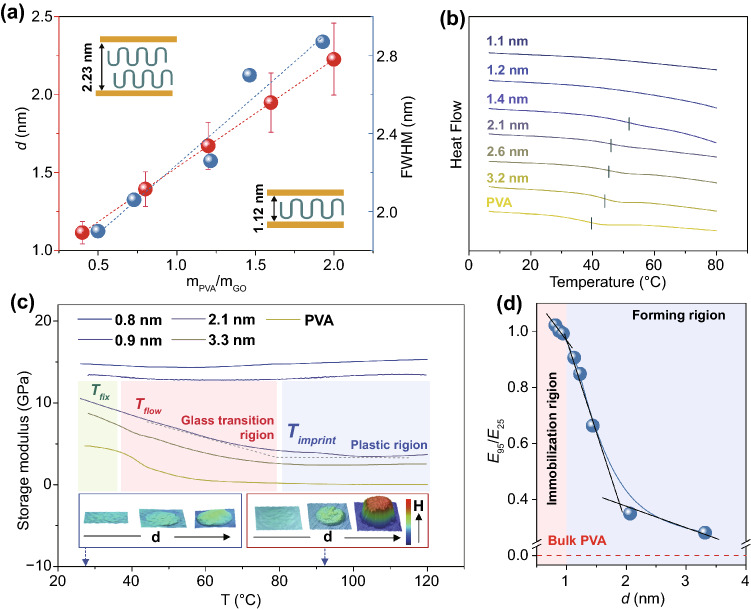
**a** Plots of the interlayer spacing and FWHM versus the weight ratio between PVA and GO. The insets presents the comparison between 1.12 nm (*m*_PVA_/*m*_GO_ = 0.4) and 2.23 nm (*m*_PVA_/*m*_GO_ = 2). **b** Glass-transition temperature measurement of Pi-GOS with different interlayer spacing. **c** Curves of storage modulus versus temperature with increasing interlayer spacing. The insets are the deformation abilities of forming structure versus *d* at 25 °C (blue) and 95 °C (red). **d** A plot of the ratio between storage modulus at 95 °C (E_95_) and that at 25 °C (E_25_) versus interlayer spacing of Pi-GOS

We investigated the thermodynamic behavior of intercalated polymer through XRD and dynamic mechanical analysis (DMA). The peak shift in XRD patterns indicates the expansion of interlayer spacing (*d*) with the increasing weight ratio of PVA to GO (*α*) and the interlayer spacing remains stable after long-term storage (Figs. 2a and S5). We detected that *d* and *α* follow a linear correlation as:

1 $$d = 0.695\alpha + 0.837$$

Meanwhile, the full width at half maximum (FWHM) obtained through XRD pattern increases with *α*, which means the decreasing orientation of GO sheets. According to Eq. (1), the thermal transition behavior can be directly connected with interlayer spacing. DSC tests revealed that *T*_*g*_ of Pi-GOS appears negative correlation with interlayer spacing and presents an increment of 12.3 °C. Meanwhile, the *T*_*g*_ disappears when *d* drops to 1.2 nm (Fig. 2b). The shift of *T*_*g*_ is caused by the increase in surface interactions between polymer and GO sheets, which suppresses the mobility of polymer chains [22, 37].

DMA tests revealed a similar transition in storage modulus of Pi-GOS as the interlayer spacing increases. For the Pi-GOS with *d* above 1.2 nm, the storage modulus manifests the second-order phase transition around the glass-transition temperature range as is stated above (Fig. 2c), compared with the stable and high storage modulus of neat GO solids [38]. The drastic decrease in storage modulus is attributed to activate softening of polymer matrix. In the *T*_fix_ region, PVA is in the glassy state and the whole film is brittle, which is failed to compression molding at any level of spacing. In the *T*_flow_ range, cooperative motions of polymer chains emerge above *T*_*g*_, resulting in a rapid decrease in storage modulus [39, 40]. When the temperature continually increases, all PVA chains transform into the rubbery state and the storage modulus stays low and steady, therefore, this region is chosen for film forming. We set 95 °C as the processing temperature to avoid the reduction of GO and decomposition of polymer as well as ensure that the polymer chain is fully activated. Furthermore, the forming ability is strongly restrained while *d* was narrowed down to a certain value. Considering the relationship between the ratio of storage modulus at 95 (*E*_*95*_) to 25 (*E*_*25*_) °C and *d*, we have detected a negative correlation curve with changing fit slope (Fig. 2d). When the spacing of layers descends to 1.1 nm or below, the ratio of *E*_*95*_ to *E*_*25*_ is close to 1. This stable storage modulus region indicates an immobilization state of film. Along with the increasing *d*, the mobility of polymer segments is activated and the thermal plasticity of Pi-GOS emerges. According to these observations, we thus used Pi-GOS with a *d* of 1.4 nm (50% content of PVA) as raw material for following forming.

**Fig. 3:**
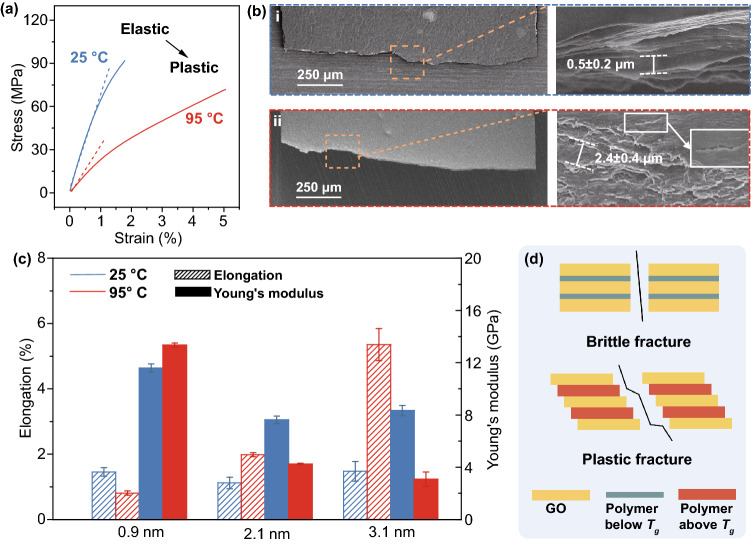
**a** Typical stress–strain curves of Pi-GOS (*d* = 3.1 nm) at 25 °C (blue) and 95 °C (red). **b** SEM images of transverse cross section morphology of Pi-GOS (*d* = 3.1 nm) after tensile breaking at 25 °C (top) and 95 °C (bottom). **c** Young’s modulus and elongation of Pi-GOS with different *d*-spacing at 25 °C (blue) and 95 °C (red). **d** Schematics of brittle tensile fracture of Pi-GOS below *T*_*g*_ and plastic tensile fracture above *T*_*g*_

To evaluate the intrinsic deformability of thermoplastic forming, we investigated the mechanic behavior of Pi-GOS. Tensile test is used to analyze the brittle-to-ductile transition of Pi-GOS with different interlayer spacings (Figs. 3a and S6a, b). The stress–strain curve of Pi-GOS measured through DMA exhibits an obviously elastic (25 °C) to plastic transition (95 °C) with a 150% increase in breakage elongation. The transition can be rationalized as the expanding *d* weakens the van der Waals interaction between GO sheets by intercalated polymer, so that the chain segments of PVA are able to move above *T*_*g*_ [15, 34, 41]. Further, Pi-GOS exhibits typical elastic deformation regardless of *d* at room temperature (Fig. 3c). The mechanical properties remain stable after cooling from processing temperature (Fig. S6c). Given the same *d* (>1.2 nm), the failure strain is enhanced when temperature increasing to 95 °C, which implies a prominent assistance provided by the motion of polymer chains and the impact becomes prominent when the contents of PVA gradually increased. While *d* is narrowed down to 1.2 nm or below, for instance 0.9 nm, the elongation shows a diminution due to the frozen PVA chains. Meanwhile, the downward trend of Young’s modulus caused by both temperature and interlayer space can be interpreted as decreasing percentage of “bricks” (GO) and the motion of “mortar” (PVA segments). A microscopic contrast of activated sheet sliding under tension at room temperature and imprinting temperature is observed through fracture morphology analysis by SEM (Fig. 3b), which is consistent with the schematic of Fig. 3d. For delamination of GO sheets under 95 °C, the fracture exhibits a winding appearance and the average slippage width is about 2.4 μm, which signifies plastic deformation (Fig. 3b-(i)) [42]. By contrast, at room temperature (25 °C), the Pi-GOS presents a straight fracture across the paper and the average slippage width is about 0.5 μm, denoting a brittle fracture behavior without thermal transition (Fig. 3b-(ii)). These results reveal that the brittle-to-ductile transition of Pi-GOS only appears when the interlayer spacing and processing temperature both satisfy the conditions.

### Multi-Scale Forming of Pi-GOS with Sub-Micro Precision

**Fig. 4:**
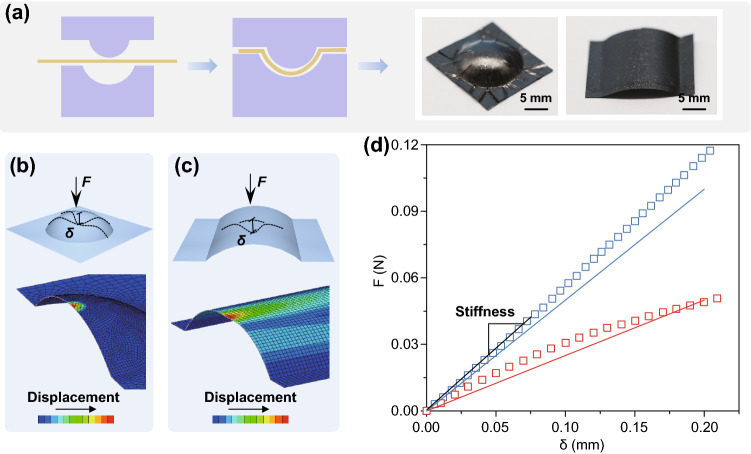
**a** Thermoplastic forming process of Pi-GOS from flat papers into stereostructures with positive curvatures. **b** FEA simulation results of indentation test for spherical and **c** cylindrical shells molded of Pi-GOS with same thickness (32 μm). **d** Force–displacement curves of spherical shell (blue square points) and cylindrical shell (red square points). Solid lines correspond to the fitting results

The materials with curved geometry usually possess enhanced mechanical stability compared to that of original flat sheets [43]. However, the inherent brittleness of graphene oxide solids confines the processing of forming GO composite film into stereo and curved shells [3]. In our work, the thermoplasticity of bulk Pi-GOS enables the fabrication of three-dimensional structures with the similar shaping techniques to polymer and metal products (Figs. 4a and S7). Starting from the flat papers, a spherical shell with positive Gaussian curvature and a cylinder with zero Gaussian curvature were shaped (Fig. 4b, c). The rigidity of curved shells was probed through indentation stiffness test carrying out by applying a point force in the convex surface center. The final force–displacement curves present the different bearing capacities between spherical and cylindrical shapes and are in good agreement with the finite element analysis (FEA) results (Fig. 4d). The stiffness (*K*) of the spherical shell, defined as the slope of the force–displacement curves, is higher than that of cylinder shell (Fig. S8), following the deduction as *K*_cylinder_ ~ *K*_spherical shell_ (*t/R*) ^1/2^ <  < *K*_spherical shell_, where *t* is the thickness and *R* is the principal radius of curvature (*t/R* <  < 1) [43–45]. The simulation results display the deformations like a mirror-buckling along a circular rage in spherical shell and a smooth-edge dimple formed with two d-cones linked by an inverted ridge in cylindrical one [43, 46].

**Fig. 5:**
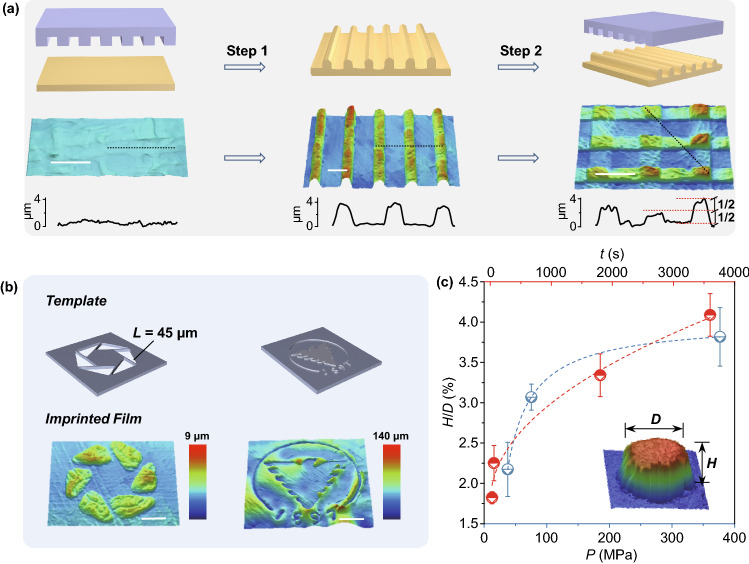
**a** Representation of multi-imprinting process from flat to micro-pattern stages. Scale bar, 50 μm. **b** Surface patterns on Pi-GOSs through micro imprinting: simple hexagon formed by triangles (left), embossment of the ZJU logo (right). Scale bars, 50 μm (left), 2 mm (right). **c** The curve of height-diameter ratio versus pressure and time. Dashed lines correspond to the fitting results and points represent experiment results (error bars are based on the standard error of the mean)

We used thermo-imprinting to fabricate micro-scale structures on the surface of Pi-GOS and the process can be double-imprinting. As shown in Fig. 5a, the composite solids can be patterned into micro-patterns by templates with corresponding size and even changeable through a programmable imprinting process. This result shows the potential of generating complex structures through one-sized template [47]. Along with the altering structure of hollow part of templates, a series of simple patterns were fabricated on the surface of composite film, including axial and central symmetry (Figs. 5b, S9 and S10). Meanwhile, complex patterns, such as the badge with varied line width, can also be generated with high fidelity. These flexible designs provide a new strategy to fabricate graphene-based solids as polymers and metals.

We further analyzed the influence factors in the height (*H*) of imprinting patterns. While the imprinted shape is settled as circle with constant diameter (*D*), the aspect ratio (*H/D*) denotes the shaping capability. For an immobile interlayer space (1.4 nm) of Pi-GOS, the imprinting pressure (${\sigma }_{s})$ and time (*t*) follow a simplified classic Norton–Bailey’s creep power law [48]:

2 $${\raise0.7ex\hbox{$H$} \!\mathord{\left/ {\vphantom {H D}}\right.\kern-\nulldelimiterspace} \!\lower0.7ex\hbox{$D$}} = B_{1} + B_{2} \sigma_{s}^{n} t^{m}$$

where *B*_*i*_ (*i* = 1, 2) are constants related to diameter, material properties and processing temperature (Fig. 5c). At a pressure of 188 MPa, the *H/D* is a function of imprinting time and the value of *m* is about 0.48. Meanwhile, the absolute value of *n* in the formula is approximately 0.96 at a fixed pressing time (5 min).

**Fig. 6:**
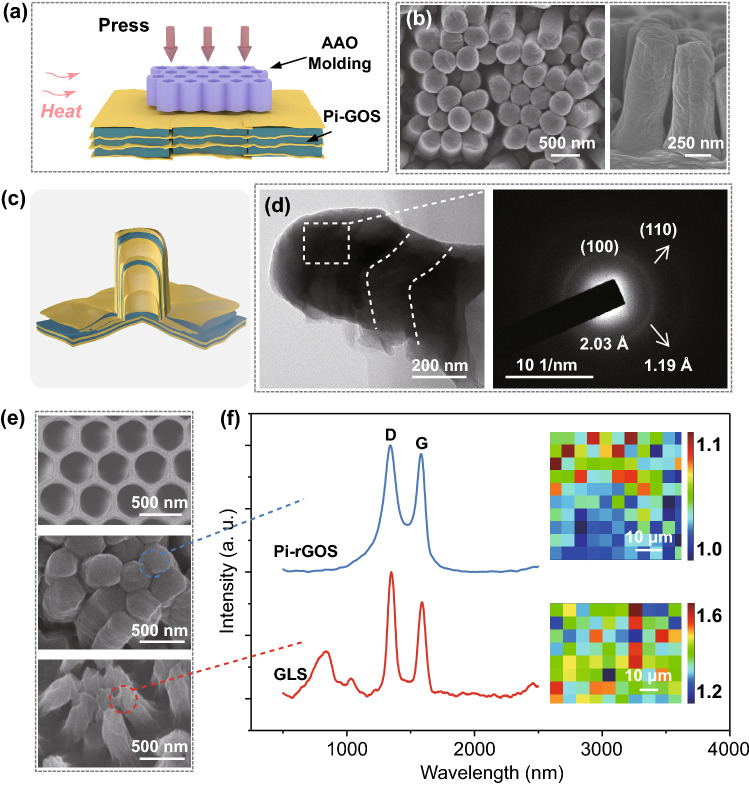
**a** Schematic diagram of thermal nanoimprinting process with through-hole AAO template. **b** SEM observations, **c** structural representation and **d** TEM images of the exterior and interior manufacture of nanorods. **e** SEM images of AAO template (top), imprinted Pi-rGOS nanopillars (middle) and heat reduced GLS nanopillars (bottom). **f** Raman spectra of Pi-rGOS and GLS. The insets are Raman mapping analysis of Pi-rGOS (up) and GLS (bottom) by considering the intensity ratio of D and G band

In addition to forming macro or micro-scale structures, nanorods with higher resolution down to 360 nm can also be generated by thermo-processing. We chose nanoporous anodic aluminum oxide (AAO) membrane with 360 nm apertures as template (Fig. 6a). After hot-pressing, the forming film was then chemical reduced and demolded. Because the forming precision is down to nanoscale, the formed solid becomes difficult to remove from the mold. The templates need to be dissolved and are therefore disposable, which can be developed in future works. Finally, the membrane with nanopillars on the surface was constructed. The nanorods are solid and perpendicular to the substratum which is formed with laterally arrayed lamella, as shown in the SEM images (Fig. 6b). The interior structure of the nanopillars is profiled in the model as shown in Fig. 6c. Under pressure at high temperature, GO sheets and PVA chains are squeezed into nanoholes and then form a hump wrapped by GO sheets when cooling down to room temperature. This solid structure indicates that the Pi-GOS fills the pores via capillary wetting because the interfacial energy between Pi-GOS and AAO template is comparatively weak [49]. The transmission electron microscopy (TEM) inspection also exhibits sawback GO sheets laying along the white dotted line inside rod and the electron diffraction pattern of the pillar wall area confirms the existence of turbostratic graphene layers outside the rod, dictated as a broaden (002) diffraction ring (Fig. 6d) [50].

Figure 6e shows that the surface topography of imprinted polymer-intercalated reduced GO solid (Pi-rGOS) is well consistent with templates. We observed that the nanorods are consisted of reduced graphene oxide sheets, exhibiting the characteristic Raman peaks at ∼1348 and ∼1585 cm^−1^ (Figs. 6f and S11) [51]. The band at ~ 2922 cm^−1^ in the spectrum results from the strongest C-H stretching of PVA [52]. After annealing treated at 1400 °C to remove PVA, the surface topography of graphene layered solid (GLS) appears as shrunken rods and the ratio of D to G peak increased from 1.0–1.1 to 1.2–1.6 (Fig. 6f). The mappings of D-G peak ratio show a homogenous distribution of imprinting structure. And after further reduction at 3000 °C, this solid possesses similar structure and properties compared with raw GO film treated in the same fashion (GF) (Figs. S12 and S13).

### Surface Properties of Nanopillar Array

**Fig. 7:**
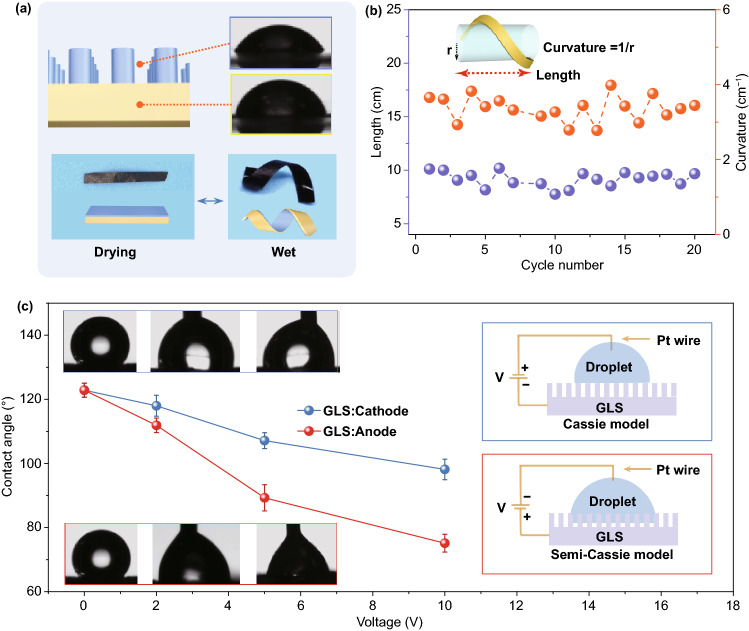
**a** Schematic illustrations of Janus imprinted Pi-rGOS with different static contact angle on each side (top) and the reversible morphing behavior of Pi-rGOS (bottom). **b** Plots of length and curvature of Janus film versus cycle number during multiple wetting tests. **c** The change of apparent contact angle with a function of voltage when the GLS was settled as cathode (blue) and anode (red), respectively, and their corresponding Cassie and Semi-Cassie contact model

The periodic structure on Pi-rGOS by nanoimprinting engineering greatly extends the surface properties of GO-based materials. The Pi-rGOS with one-side nanorods performs as Janus properties, which exhibits different water-contact angles of 81° on plain side and 56° on imprinted side (Fig. 7a). A straight film gradually bends when immersed into water and transforms into spiral shape quickly (a few seconds) (Figs. 7a and S14a). This Janus wettability promotes an implementation of water-induced actuator due to larger capillary forces [53] of nanorods, which is served as a driving force for complex deformation (Fig. S14b, c). The stable variation of ultimate curvature and length during 20 cycles of actuated deformation (Fig. 7b) demonstrates that the Pi-rGOS actuator possesses high reversibility and repeatability, which exhibits great potential in soft robotics and smart electronics.

We also found the surface wettability of the GLS can be electrically adjustable by the effect of DC bias. To ensure the electrical conductivity, we chose heat-annealed GLS as a demo. A Pt wire was inserted into water droplet to establish electrical contact. Once the membrane is set as anode, the contact angle shows a sharp decrease from 123° to 75° as the droplet gradually sinking into membrane, which is corresponding to transformation from Cassie model (hydrophobic) to Semi-Cassie model (hydrophilic) (Fig. 7c). By contrast, the variation is weaker when a negative bias is applied to the GLS. Regardless of polarity, the wetting mechanism can be explained by the decline of solid–fluid interfacial tension. While the polarity-induced difference in wettability is resulted from the oxidation of a small minority of graphene on the surface [54, 55]. In addition, the droplet pumping speed shows a dependence on bias and the decrease in contact angle is much quickly at higher voltage, which is originated from the faster electrochemical oxidation speed (Fig. S15). Depending on the electrically adjustable wettability of GLS, we designed a sandwiched device for water transportation (Fig. S16). A water droplet (10 μL) was placed between two identical imprinted membranes and stayed on one side when no bias applied, which can be transferred to anode side via applying positive voltage of 10 V.

## Conclusions

In this study, we developed a solvent-free thermoplastic forming of GO solid by polymer intercalation with high precision. We found that the thermoplasticity of Pi-GOS is fulfilled when the interlayer spacing exceeds 1.4 nm, which is the criterion for activation of intercalated polymer chain. Based on the thermoplasticity, we proposed the hot-shaping and thermal imprinting methods to process flat solids into rich structural forms with multiscale stereo attributes, ranging from macro to nanolevel (360 nm). Those complex deformations modify mechanical property and responsiveness of Pi-GOS and the thermoplastic forming materials remain good electrical as well as thermal conductivity after thermal annealing. The solvent-free thermoplastic forming approach provides new forming technology of GO materials and other layered materials, and further broadens the applications in versatile devices.

## Supplementary Information

Below is the link to the electronic supplementary material.

Supplementary file1 (PDF 8783 kb)
