# Supplementary material for: Precise Thermoplastic Processing of Graphene Oxide Layered Solid by Polymer Intercalation
Source: Nanomicro Lett. 2021 Dec 4;14:12. doi: 10.1007/s40820-021-00755-8 (PMC8643290; doi:10.1007/s40820-021-00755-8)
Supplement: Supplementary file 1 — Supplementary file1 (PDF 8783 kb) [file 40820_2021_755_MOESM1_ESM.pdf]

Nano-Micro Letters

Supporting Information for

# Precise Thermoplastic Forming of Graphene Oxide Layered Solid by Polymer Intercalation

*Zeshen Li[1], Fan Guo[1,2*], Kai Pang[1], Jiahao Lin[1], Qiang Gao[3], Yance Chen[1], Dan Chang[1], Ya Wang[1], Senping Liu[1], Yi Han[4], Yingjun Liu[1], Zhen Xu[1*], Chao Gao[1*]*

[1]MOE Key Laboratory of Macromolecular Synthesis and Functionalization, Department of Polymer Science and Engineering, Key Laboratory of Adsorption and Separation Materials & Technologies of Zhejiang Province, Zhejiang University, 38 Zheda Road, Hangzhou 310027, P. R. China.

[2]National Special Superfine Powder Engineering Research Center, Nanjing University of Science and Technology, 1 Guanghua Road, Nanjing 210094, P. R. China.

[3]School of Mechanical Engineering, Southeast University, Nanjing, Jiangsu 211189, P. R. China.

[4]Hangzhou Gaoxi Technology Co. Ltd., Liangzhu, Yuhang District, Hangzhou, 311113, P. R. China.

* Corresponding author. E-mail: fanguo@njust.edu.cn; zhenxu@zju.edu.cn; chaogao@zju.edu.cn

## Supplementary Figures

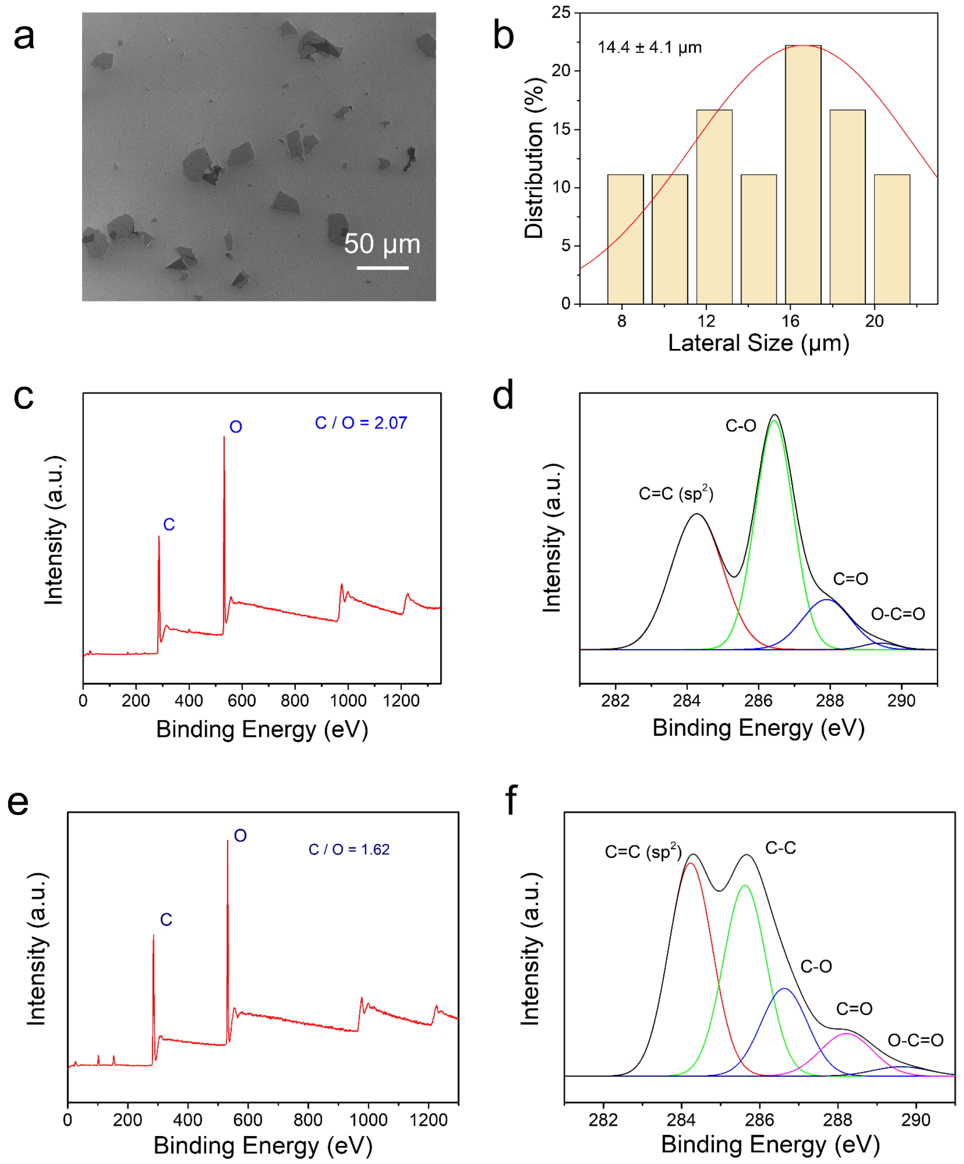


**Fig. S1** Characterization data of GO sheets, GO film and Pi-GOS. (**a-b**) SEM images and size distribution of GO sheets with lateral size around 14.4 μm. (**c-d**) XPS spectra for full elements (**c**) and C1s (**d**) of GO film, divided into the four peaks: C=C skeleton and many oxygen-containing peaks (C-O, C=O and O-C=O). (**e-f**) XPS spectra for full elements (**e**) and C1s (**f**) of Pi-GOS (50% PVA), divided into the five peaks: C=C and C-C skeleton and many oxygen-containing peaks (C-O, C=O and O-C=O).

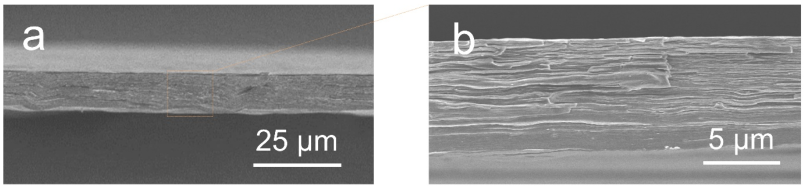


**Fig. S2** The SEM images of Pi-GOS with lamellar structure.

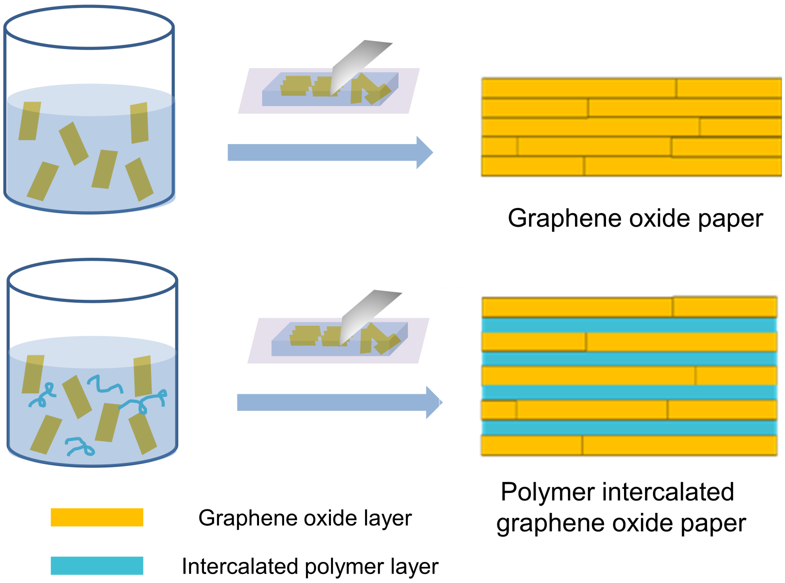


**Fig. S3** The illustration of the polymer intercalating process and lamellar structures.

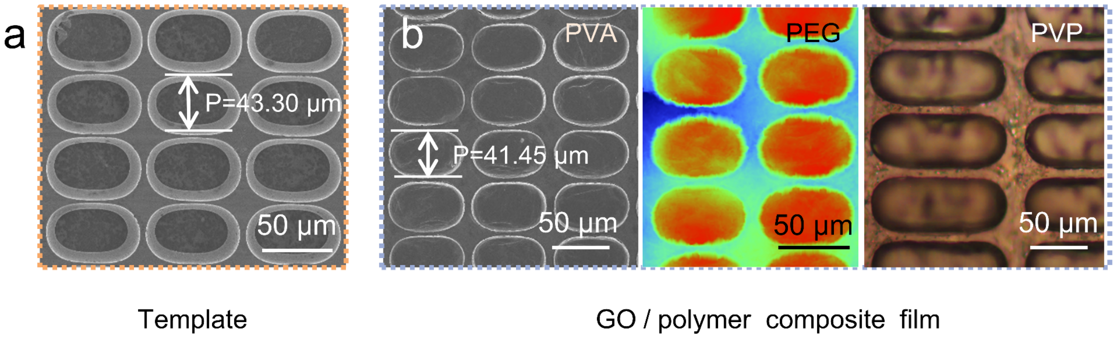


**Fig. S4** Demonstration of the formability of Pi-GOS with different kinds of polymer. (**a**) SEM image of template. (**b-d**) The surface patterns on Pi-GOS composed of PVA (**b**), PEG (**c**) and PVP (**d**).

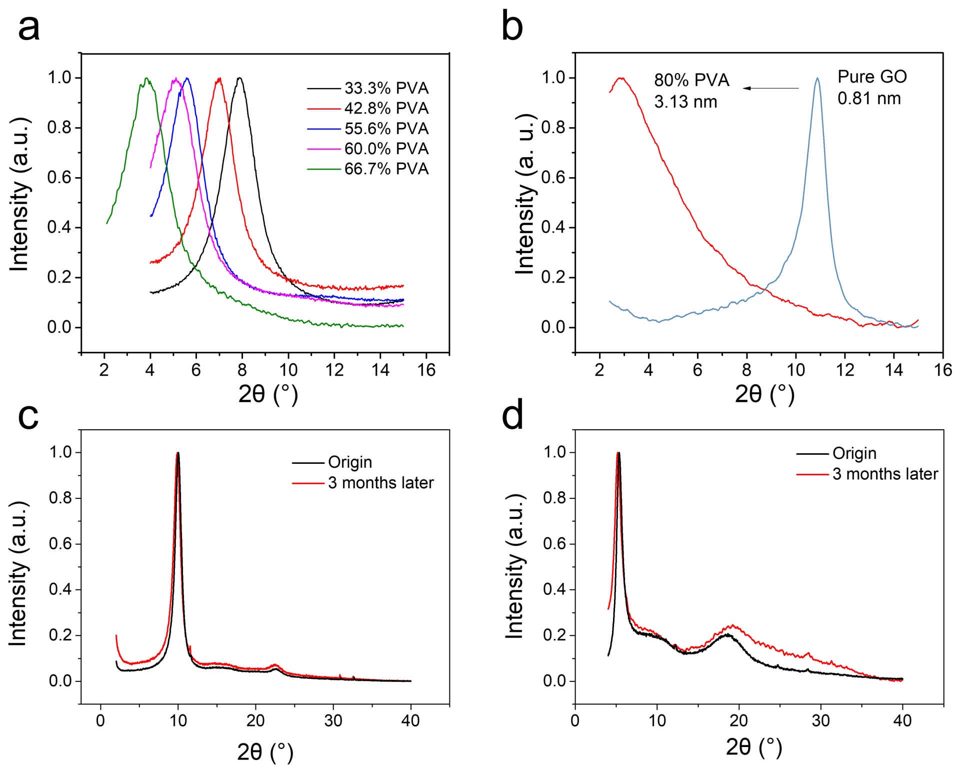


**Fig. S5** XRD patterns of (a) Pi-GOS with different PVA contents, (b) the peak shift between pure GO film and composite film (80% PVA), (c) pure GO film and (d) Pi-GOS (50% PVA) now and after three months.

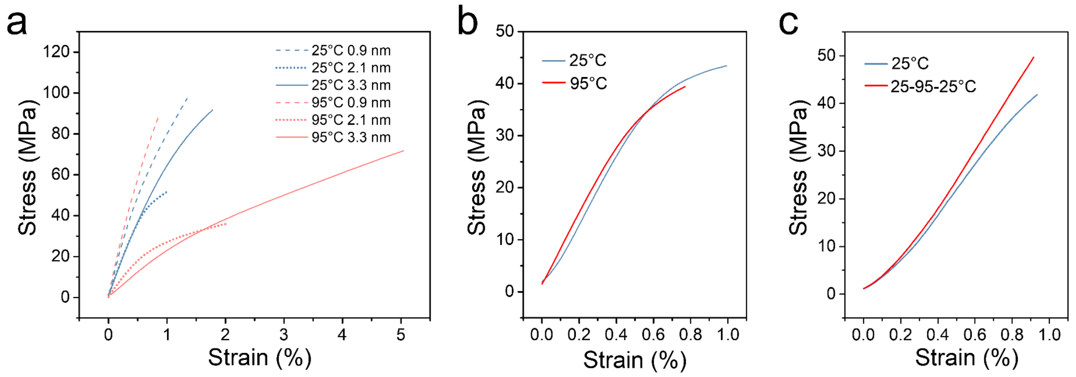


**Fig. S6** Typical stress–strain curves of (**a**) Pi-GOS with different *d*-spacing, (**b**) pure GO film under different temperature and (**c**) Pi-GOS ($d$ = 1.4 nm) under room temperature before and after annealing treatment.

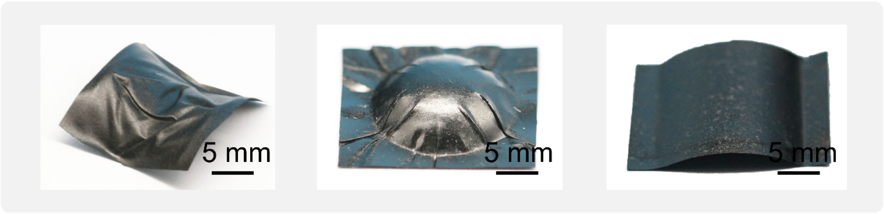


**Fig. S7** The forming Pi-GOS with different Gauss curvatures.

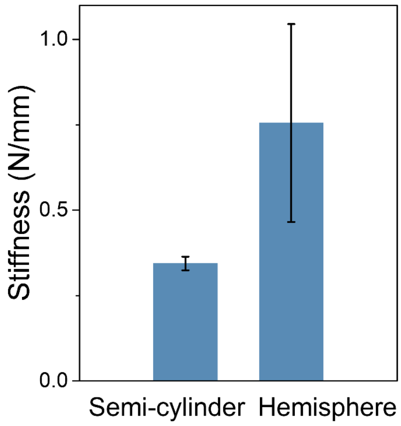


**Fig. S8** Summary of stiffness of shells with different Gauss curvatures.

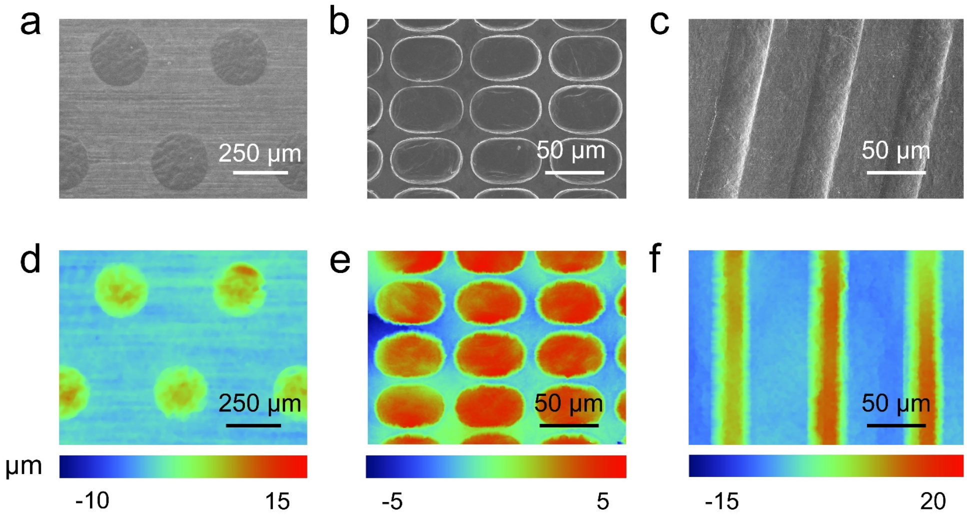


**Fig. S9** Simple surface patterns on Pi-GOS through micro imprinting: (**a-c**) SEM images; (**d-f**) 3D-profile images.

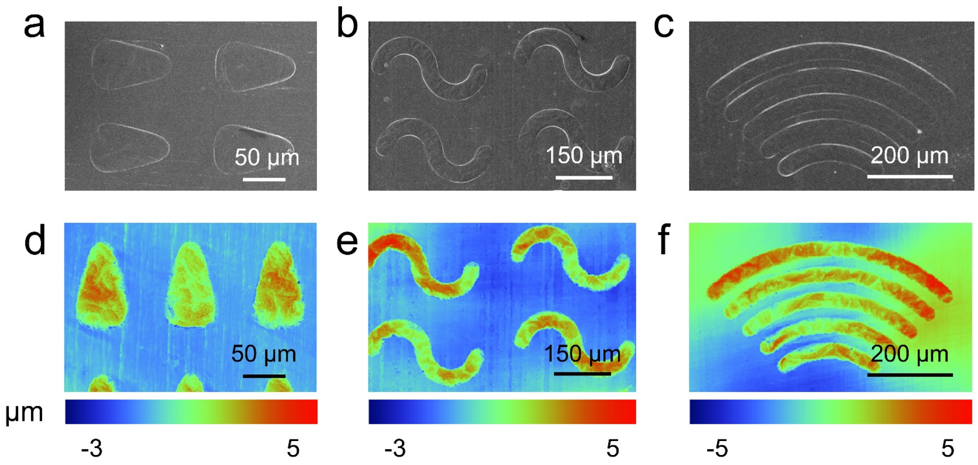


**Fig. S10** Complex surface patterns on Pi-GOS through micro imprinting: (**a-c**) SEM images; (**d-f**) 3D-profile images.

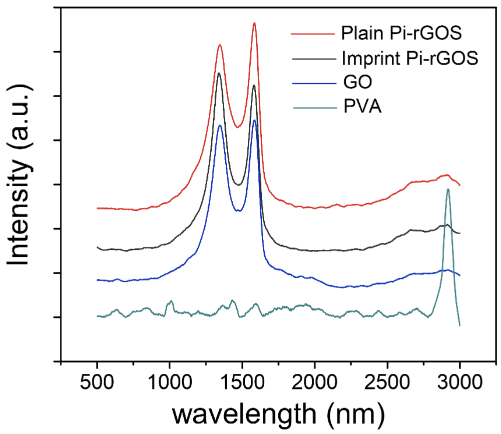


**Fig. S11** Raman spectra of pure GO and PVA film and Pi-rGOS before and after imprinted.

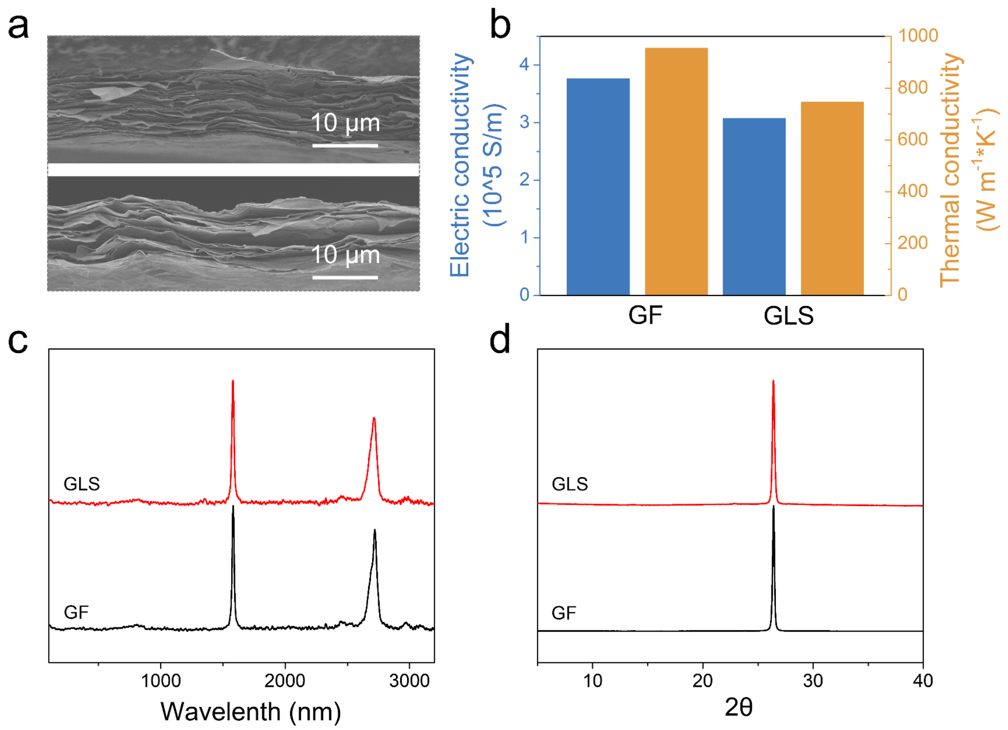


**Fig. S12** Structure and properties characterization of GF and GLS. (**a**) The SEM images of GF (top) and GLS (bottom) with lamellar structure. (**b-d**) Electric and thermal conductivity (**b**), Raman spectrum (**c**) and XRD patterns (**d**) of GF and GLS.

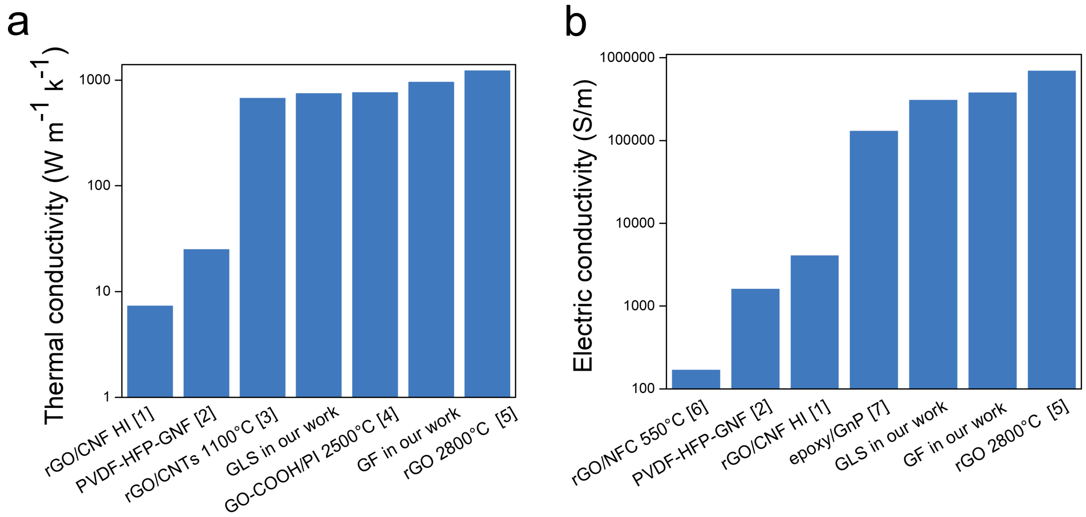


**Fig. S13** Comparison of (**a**) thermal and (**b**) electric conductivity of our produced materials with other similar films in the literatures.

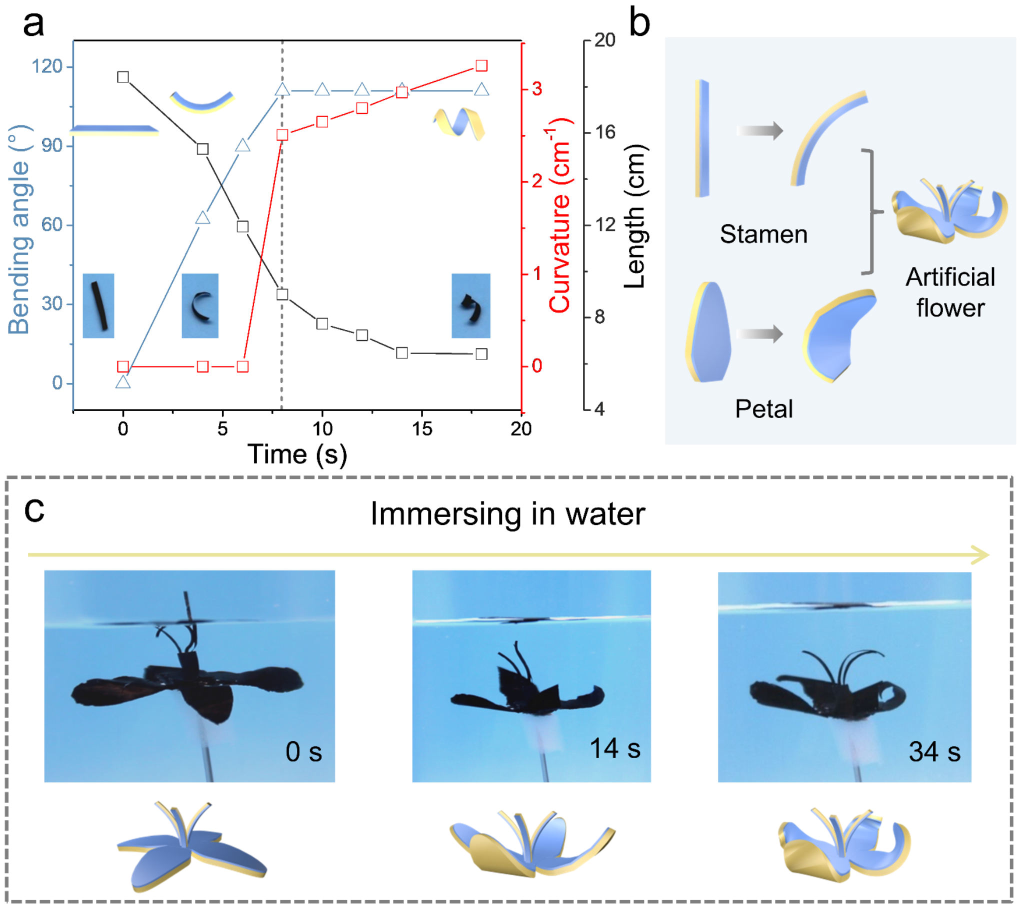


**Fig. S14** The morphing behavior of Pi-rGOS. (**a**) Curves of bending angle, curvature and normalized length versus the immersing time. (**b-c**) Schematic illustration and digital images of the folding process of an artificial flower assembled from the imprinted Pi-rGOS immersing in water.

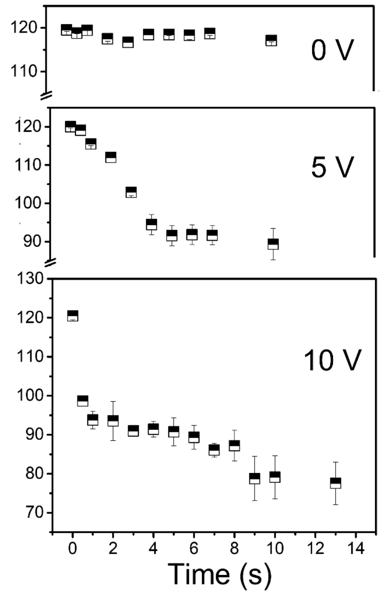


**Fig. S15** Apparent contact angle variation of a water droplet on imprinted membrane with positive bias of 0, 5, and 10 V respectively.

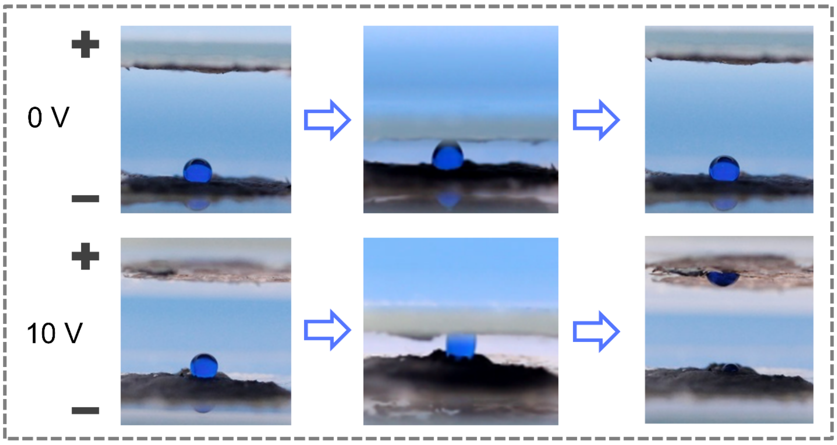


**Fig. S16** Water droplet transportation response with imprinted membrane electrodes as both anode and cathode: without bias (top); the top film electrode as anode (bottom). The water droplet can be pumped from cathode to anode side by the effect of voltage while stays stable with no bias applied.

## Supplementary References

[S1] W.X. Yang, Z.D. Zhao, K. Wu, R. Huang, T.Y. Liu et al., Ultrathin flexible reduced graphene oxide/cellulose nanofiber composite films with strongly anisotropic

thermal conductivity and efficient electromagnetic interference shielding. J. Mater. Chem. C **5**, 3748--3756 (2017). https://doi.org/10.1039/C7TC00400A

[S2] A.A. Tarhini, A.R. Tehrani-Bagha, Graphene-based polymer composite films with enhanced mechanical properties and ultra-high in-plane thermal conductivity. Compos Sci Technol **184**, 107797 (2019). https://doi.org/10.1016/j.compscitech.2019.107797

[S3] G.J. Yuan, J.F. Xie, H.H. Li, B. Shan, X.X. Zhang et al., Thermally reduced graphene oxide/carbon nanotube composite films for thermal packaging applications. Materials **13**(2), 317 (2020). https://doi.org/10.3390/ma13020317

[S4] L.R. Ma, Y.X. Wang, X.D. Xu, Y.Y. Wang, C.G. Wang, Structural evolution and thermal conductivity of flexible graphite films prepared by carboxylic graphene/polyimide. Ceram. Int. **47**, 1076–1085 (2021). https://doi.org/10.1016/j.ceramint.2020.08.223

[S5] X.D. Zhang, Y. Guo, Y.J. Liu, Z. Li, W.Z. Fang et al., Ultrathick and highly thermally conductive graphene films by self-fusion. Carbon **167**, 249-255 (2020). https://doi.org/10.1016/j.carbon.2020.05.051

[S6] J.J. Chen, H.L. Li, L.H. Zhang, C. Du, T. Fang et al., Direct reduction of graphene oxide/nanofibrillated cellulose composite film and its electrical conductivity research. Sci. Rep. **10**, 3124 (2020). https://doi.org/10.1038/s41598-020-59918-z

[S7] Q.S. Meng, H. Wu, Z.H. Zhao, S. Araby, S.W. Lu et al., Free-standing, flexible, electrically conductive epoxy/graphene composite films. Compos. Part A **92**, 42–50 (2017). http://doi.org/10.1016/j.compositesa.2016.10.028
